# Dexamethasone, Prednisolone, and Methylprednisolone Use and 2-Year Neurodevelopmental Outcomes in Extremely Preterm Infants

**DOI:** 10.1001/jamanetworkopen.2022.1947

**Published:** 2022-03-11

**Authors:** Mihai Puia-Dumitrescu, Thomas R. Wood, Bryan A. Comstock, Janessa B. Law, Kendell German, Krystle M. Perez, Semsa Gogcu, Dennis E. Mayock, Patrick J. Heagerty, Sandra E. Juul

**Affiliations:** 1 Division of Neonatology, Department of Pediatrics, University of Washington, Seattle; 2 Department of Biostatistics, University of Washington, Seattle; 3 Division of Neonatology, Department of Pediatrics, Wake Forest University School of Medicine, Winston-Salem, North Carolina

## Abstract

**Importance:**

Practice variability exists in the use of corticosteroids to treat or prevent bronchopulmonary dysplasia in extremely preterm infants, but there is limited information on longer-term impacts.

**Objective:**

To describe the use of corticosteroids in extremely preterm infants and evaluate the association with neurodevelopmental outcomes.

**Design, Setting, and Participants:**

This cohort study was a secondary analysis of data from the Preterm Erythropoietin Neuroprotection (PENUT) randomized clinical trial, conducted at 19 participating sites and 30 neonatal intensive care units (NICUs) in the US. Inborn infants born between 24 0/7 and 27 6/7 weeks gestational age between December 2013 and September 2016 were included in analysis. Data analysis was conducted between February 2021 and January 2022.

**Exposures:**

Cumulative dose of dexamethasone and duration of therapy for dexamethasone and prednisolone or methyl prednisolone were evaluated.

**Main Outcomes and Measures:**

Demographic and clinical characteristics were described in infants who did or did not receive corticosteroids of interest and survived to discharge. Neurodevelopmental outcomes at 2 years of age were evaluated using the Bayley Scales of Infant Development–Third Edition (BSID-III) at corrected age 2 years.

**Results:**

A total of 828 extremely preterm infants (403 [49%] girls; median [IQR] gestational age, 26 [25-27] weeks) born at 19 sites who survived to discharge were included in this analysis, and 312 infants (38%) were exposed to at least 1 corticosteroid of interest during their NICU stay, including 279 exposed to dexamethasone, 137 exposed to prednisolone or methylprednisolone, and 79 exposed to both. Exposed infants, compared with nonexposed infants, had a lower birth weight (mean [SD], 718 [168] g vs 868 [180] g) and were born earlier (mean [SD] gestational age, 25 [1] weeks vs 26 [1] weeks). The median (IQR) start day was 29 (20-44) days for dexamethasone and 53 (30-90) days for prednisolone or methylprednisolone. The median (IQR) total days of exposure was 10 (5-15) days for dexamethasone and 13 (6-25) days for prednisolone or methylprednisolone. The median (IQR) cumulative dose of dexamethasone was 1.3 (0.9-2.8) mg/kg. After adjusting for potential confounders, treatment with dexamethasone for longer than 14 days was associated with worse neurodevelopmental outcomes, with mean scores in BSID-III 7.4 (95% CI, –12.3 to –2.5) points lower in the motor domain (*P* = .003) and 5.8 (95% CI, –10.9 to –0.6) points lower in the language domain (*P* = .03), compared with unexposed infants.

**Conclusions and Relevance:**

These findings suggest that long duration and higher cumulative dose of dexamethasone were associated with worse neurodevelopmental scores at corrected age 2 years. Potential unmeasured differences in the clinical conditions of exposed vs unexposed infants may contribute to these findings. Improved standardization of treatment and documentation of indications would facilitate replication studies.

## Key Points

**Question:**

What are the patterns of dexamethasone and prednisolone and methylprednisolone use in extremely preterm infants and are their use associated with 2-year outcomes?

**Findings:**

In this cohort study including 828 extremely preterm infants from a multicenter randomized clinical trial, 38% of infants were treated with corticosteroids, with exposure rates decreasing as gestational age at birth increased. More infants were treated with dexamethasone, and exposure was shorter and earlier compared with prednisolone or methylprednisolone; longer duration of treatment with dexamethasone, but not prednisolone or methylprednisolone, was associated with worse neurodevelopmental outcomes at corrected age 2 years.

**Meaning:**

These findings suggest that significant practice variation in postnatal corticosteroids treatment was common and that limiting exposure to dexamethasone may be associated with minimizing adverse neurodevelopmental outcomes.

## Teaser

This cohort study describes the use of corticosteroids in extremely preterm infants and the association of corticosteroid exposure with neurodevelopmental outcomes at 2 years.

## Introduction

Bronchopulmonary dysplasia (BPD) is a common complication of prematurity, affecting 20% to 75% of infants born before gestational age (GA) 29 weeks.^1,2,3^ BPD is associated with delayed brain maturation and diffuse white matter anomalies that are associated with increased risk of neurodevelopmental impairment.^4,5,6^ Postnatal corticosteroids have been used to prevent and treat BPD for more than 50 years in multiple trials involving thousands of infants.^7,8,9^ Dexamethasone, a long-acting glucocorticoid agonist, is 1 of the top 20 medications used in extremely preterm infants,^10,11^ primarily to prevent or treat developing BPD. Multiple small trials and meta-analyses have demonstrated that short courses of low-dose dexamethasone may be useful for earlier weaning and extubation of infants undergoing mechanical ventilation at highest risk of BPD.^8,9,12^ However, the administration of dexamethasone to prevent BPD, especially in the first week of life, has been associated with increased risk for adverse effects, including cognitive, speech, and learning impairments, as well as cerebral palsy (CP).^13,14,15,16,17,18,19,20,21,22,23,24,25^ Routine dexamethasone therapy in the first week of life is not recommended.^26,27,28^ Currently, most clinicians prescribe low-dose, late-initiated dexamethasone^29^ of shorter duration than what was previously used^28,30^ to maximize benefits and minimize potential risks. Prednisolone and methylprednisolone are synthetic glucocorticoids with similar potency that are used interchangeably in practice to prevent or treat BPD. Their effectiveness has only been evaluated retrospectively.^31,32^

Given inconclusive evidence regarding the association between dexamethasone and prednisolone or methylprednisolone initiation, dosage, length of exposure, and subsequent outcomes of extremely preterm infants, we have performed an analysis using data from a large contemporary multicenter trial with the following goals: to characterize the use of dexamethasone and prednisolone or methylprednisolone, to identify factors associated with exposure, and to estimate associations between key measures of exposure (ie, duration and dose) and 2-year neurodevelopmental outcomes.

## Methods

### Data Source and Study Population

All infants enrolled in the Preterm Erythropoietin Neuroprotection (PENUT) trial (ClinicalTrials.gov Identifier: NCT01378273) were eligible for this study.^33^ The PENUT trial was approved by institutional review boards at each site. Parental consent was obtained before or after birth, as permitted by the institutional review board at each site. This study followed the Strengthening the Reporting of Observational Studies in Epidemiology (STROBE) reporting guideline.

We collected data about maternal characteristics, pregnancy, and delivery, as well as infant characteristics, including exposure to medications and comorbidities during their neonatal intensive care unit (NICU) stay. At corrected age 20 to 33 months, infants were evaluated by certified examiners who assessed cognitive, motor, and language development with the Bayley Scales of Infant Development–Third Edition (BSID-III).^34^ Higher BSID-III scores indicated better performance, with scores of less than 85 points indicating 1 SD below the mean and less than 70 points, 2 SDs below the mean. The presence of CP was evaluated by standardized neurologic examination^35^ and graded by the Gross Motor Function Classification System^36^ on scale of 0 to 5, with 1 indicating mild CP and 2 to 5, moderate or severe CP.

The population of interest was infants exposed to dexamethasone, prednisolone, or methylprednisolone during their initial NICU course. Exposure to dexamethasone, prednisolone, or methylprednisolone was defined as having at least 1 dose of drug administered at any time during the hospital stay, and no exposure if there were no documented doses administered during that time. The length of exposure was classified by total days of exposure as approximate quartiles of number of days exposed (dexamethasone: ≤3 days, 4-7 days, 8-14 days, or >14 days; prednisolone or methylprednisolone: 1-7 days, 8-14 days, 14-28 days, or >28 days). These quantiles were constructed by determining the quantiles of exposure among the exposed and then rounding them into more clinically relevant quantiles. The association of days of exposure as a continuous variable was also examined for both classes of steroids, as well as total cumulative dose in milligrams per kilogram for dexamethasone. Number of courses and length of each course for both classes of steroid were determined based on number of consecutive days of steroid exposure, with at least 1 day of no exposure determining the start of the next course.

### Statistical Analysis

We used descriptive statistics to describe the demographic and baseline maternal and infant characteristics and exposure to corticosteroids of interest by study site. Maternal and infant variables were compared in exposed and nonexposed infants individually after adjusting for GA and study treatment group, with Bonferroni correction of *P* values for 28 comparisons (target *P* value: .05/28 = .0017). We aimed to evaluate the association between corticosteroids of interest and neurodevelopmental outcomes measured by BSID-III and CP scores. We used generalized estimating equations (GEE)^37^ with robust SEs and an independence covariance structure as the statistical model to account for potential correlation of outcomes for same-birth siblings. Specifically, we performed logistic GEE regression to examine differences in infant characteristics between those who were and were not exposed to corticosteroids. We then used linear multivariable GEE analyses using each of the 3 BSID-III scores (cognitive, motor, and language) as the outcome. We chose to conduct a sequence of outcome regression models that allow characterization of dose associations while accounting for potential confounders. We developed 3 different models to estimate the parallel associations of steroid exposure using quartiles of length of exposure (days) or total cumulative dose; model 1 was adjusted for treatment group (erythropoietin/placebo) and GA at birth in weeks; model 2 was additionally adjusted for potential confounders, ie, being small for gestational age (SGA) and level of respiratory support at 14 days (none or nasal cannula, positive pressure, or intubated); model 3 was further adjusted for level of maternal education and in-hospital severe adverse events (SAEs) that are associated with outcomes to improve the accuracy of the estimated association of steroid exposure.

Sensitivity analyses included model 3 with further adjustment for clinical site. SAEs included severe BPD (defined as respiratory support with nasal cannula or more at postmenstrual age 36 weeks), grade 2b to 3 necrotizing enterocolitis, spontaneous intestinal perforation, retinopathy of prematurity requiring intervention, and grade III to IV intracranial hemorrhage at any time during the hospital course. Infants who required any supplemental oxygen at discharge were considered to require respiratory support at discharge. We reported the estimated mean differences in BSID-III scores between groups characterized by amount of exposure, along with their 95% CIs and corresponding *P* values. Maternal and infant variables were compared in exposed and nonexposed infants individually after adjusting for GA and study treatment group, with Bonferroni correction of P values for 28 comparisons (target *P* = .05/28 = .0017). All other *P* < .05 were considered statistically significant. All *P* values were 2-sided

When examining exposure to dexamethasone or prednisolone or methylprednisolone as continuous variables by days of exposure or cumulative dose, the models included both any or no exposure as variables, as well as the days and dose of exposure. An example figure depicting the proposed relationship between days of dexamethasone exposure and BSID-III motor score is shown in eFigure 1 in Supplement 1. Finally, logistic GEE regression models were performed to examine the association between any steroid exposure and diagnosis of CP, adjusting for covariates as in model 3.

All analyses were conducted using the R statistical package version 3.6.3 (R Project for Statistical Computing). Data were analyzed between February 2021 and January 2022.

## Results

### Cohort Characteristics

**Figure 1.**
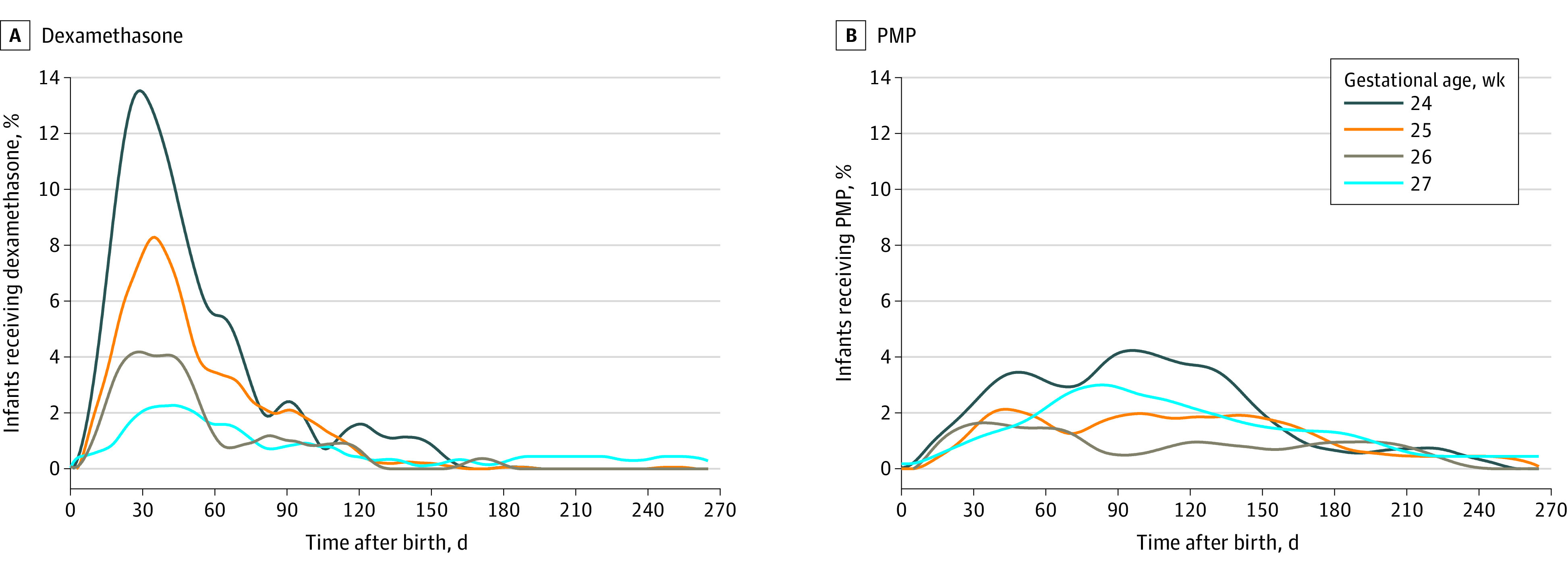
Prevalence of Exposure to Dexamethasone and Prednisolone or Methylprednisolone (PMP) Over Time, Stratified by Gestational Age at Birth The daily prevalence of exposure, defined as the number of exposed infants divided by the number that remained alive and in hospital, was plotted for each gestational age by postnatal age. Proportion of exposed infants each day was calculated based on the number of infants of that gestational age surviving to that day.

A total of 828 infants (403 [48.7%] girls; 213 [25.7%] Black infants and 535 [64.6%] White infants; median [IQR] gestational age, 26 [25-27] weeks) who survived to discharge were included in this analysis, and 312 infants (37.7%) were exposed to at least 1 corticosteroid of interest (dexamethasone and/or prednisolone or methylprednisolone) (Table 1). A total of 186 infants (22.5%) were exposed to dexamethasone, 54 infants (6.5%) were exposed to prednisolone or methylprednisolone and 72 infants (8.7%) were exposed to both. Exposed infants, compared with unexposed infants, had a lower birth weight (mean [SD], 718 [168] g vs 868 [180] g) and were born earlier (mean [SD] GA, 25 [1] weeks vs 26 [1] weeks). Exposure to corticosteroids decreased with advancing GA, from 123 of 192 infants (64.1%) born at GA 24 weeks to 43 of 223 infants (19.3%) born at GA 27 weeks. When comparing dexamethasone with prednisolone or methylprednisolone, more infants were exposed to dexamethasone than prednisolone or methylprednisolone, and this exposure occurred earlier in life (Figure 1). There were no significant differences in maternal, pregnancy, or delivery characteristics between groups, other than exposed infants being more likely to have been born by Caesarean delivery (Table 1).

**Table 1.** Demographic and Baseline Characteristics by Dexamethasone, Prednisolone, or Methylprednisolone Exposure

Characteristic	No. (%)	
	Not Exposed (n = 516)	Exposed (n = 312)
**Maternal factors**		
Race		
White	333 (62.2)	202 (37.8)
Black	130 (61.0)	83 (39.0)
Other^a^	38 (71.7)	15 (28.3)
Education		
≤High school	182 (65.7)	95 (34.3)
Some college	151 (59.4)	103 (40.6)
≥College degree	130 (62.8)	77 (37.2)
Unknown or not reported	53 (58.9)	37 (41.1)
Obesity		
No	467 (62.8)	277 (37.2)
Yes	49 (58.3)	35 (41.7)
Hypertension		
No	413 (62.1)	252 (37.9)
Yes	103 (63.2)	60 (36.8)
Prenatal care		
No	16 (66.7)	8 (33.3)
Yes	491 (62.2)	298 (37.8)
Unknown	9 (60.0)	6 (40.0)
Multiple gestation pregnancy		
No	376 (61.6)	234 (38.4)
Yes	140 (64.2)	78 (35.8)
Preterm labor		
No	185 (61.7)	115 (38.3)
Yes	331 (62.7)	197 (37.3)
Prolonged rupture of membranes		
No	384 (63.4)	222 (36.6)
Yes	132 (59.5)	90 (40.5)
Chorioamnionitis		
No	447 (62.3)	271 (37.7)
Yes	69 (62.7)	41 (37.3)
Prenatal antibiotics		
No	324 (61.6)	202 (38.4)
Yes	192 (63.6)	110 (36.4)
Prenatal steroids		
No	40 (64.5)	22 (35.5)
Yes	469 (62.2)	285 (37.8)
Unknown	7 (58.3)	5 (41.7)
Prenatal magnesium		
No	74 (57.8)	54 (42.2)
Yes	424 (63.6)	243 (36.4)
Unknown	17 (53.1)	15 (46.9)
Caesarean delivery^b^		
No	181 (69.6)	79 (30.4)
Yes	335 (59.0)	233 (41.0)
**Infant factors**		
Gestational age, wk^b^		
24	69 (35.9)	123 (64.1)
25	118 (55.9)	93 (44.1)
26	149 (73.8)	53 (26.2)
27	180 (80.7)	43 (19.3)
Sex		
Girls	265 (65.8)	138 (34.2)
Boys	251 (59.1)	174 (40.9)
Birth weight <10th percentile^b^		
No	459 (65.0)	247 (35.0)
Yes	54 (45.8)	64 (54.2)
Delayed cord clamping		
No	191 (61.0)	122 (39.0)
Yes	168 (57.3)	125 (42.7)
Unknown or not reported	156 (70.6)	65 (29.4)
Intubation or chest compressions at birth		
No	129 (77.2)	38 (22.8)
Yes	387 (58.5)	274 (41.5)
Apgar score <5 at 5 min		
No	437 (64.5)	240 (35.5)
Yes	77 (52.0)	71 (48.0)
General appearance at birth		
Well	330 (66.8)	164 (33.2)
Sick	184 (55.8)	146 (44.2)
Respiratory support at 14 d^b^		
None or low-flow nasal cannula	35 (97.2)	1 (2.8)
High-flow or positive pressure	323 (86.8)	49 (13.2)
Intubation or ventilation	158 (37.7)	261 (62.3)
Serious adverse events (ever)		
Severe sepsis		
No	491 (63.3)	285 (36.7)
Yes	25 (48.1)	27 (51.9)
NEC		
No	496 (62.7)	295 (37.3)
Yes	20 (54.1)	17 (45.9)
Severe BPD^b^		
No	255 (86.7)	39 (13.3)
Yes	261 (48.9)	273 (51.1)
SIP		
No	506 (63.9)	286 (36.1)
Yes	10 (27.8)	26 (72.2)
Severe IVH at any time during NICU stay		
No	469 (90.9)	47 (9.1)
Yes	47 (51.6)	44 (48.4)
ROP		
No	490 (64.4)	271 (35.6)
Yes	26 (38.8)	41 (61.2)
Respiratory support at discharge^b^		
No	380 (75.1)	126 (24.9)
Yes	136 (42.2)	186 (57.8)

Abbreviations: BPD, bronchopulmonary dysplasia; IVH, intraventricular hemorrhage; NEC, necrotizing enterocolitis; NICU, neonatal intensive care unit; ROP, retinopathy of prematurity; SIP, spontaneous intestinal perforation.

^a^ Other race includes American Indian or Alaska Native, Native Hawaiian or Pacific Islander, Asian, and unknown or unreported.

^b^ Indicates significant difference between exposed and unexposed infants after Bonferroni correction for 28 comparisons. Same differences between exposed and unexposed were also present when splitting the exposed into dexamethasone vs prednisolone or methylprednisolone vs both. Target *P* value was .05/28 = .0017. All models were adjusted for treatment group (erythropoietin/placebo) and gestational age.

At age 14 days, more infants who remained intubated received corticosteroids (261 of 312 infants [83.7%]) compared with the nonexposed group (158 of 516 infants [30.6%]) (*P* < .001). Similarly, at postmenstrual age 36 weeks, 273 of 312 infants (87.5%) in the exposed group vs 261 of 516 infants (50.6%) in the nonexposed group were diagnosed with severe BPD (*P* < .001). At discharge, 186 of 312 infants (59.6%) in the exposed group and 136 of 516 infants (26.4%) in the nonexposed group required respiratory support (*P* < .001).

The prevalence of reported SAEs was similar between the exposed and nonexposed groups, except for BPD, which was higher in the exposed group (Table 1). The most common SAEs in the exposed group were severe BPD and grade III to IV intracranial hemorrhage (Table 1). The overall rates of necrotizing enterocolitis and spontaneous intestinal perforation in the exposed infants were low. Exposed infants had longer hospital stays compared with nonexposed infants (mean [SD] length of stay, 136 [51] days vs 97 [36] days; *P* < .001). Estimated adjusted mean difference in length of stay for infants receiving corticosteroids after adjusting for GA and treatment group was 33 (95% CI, 26-41) days longer than nonexposed infants.

### Exposure Variables of Interest

In the exposed group, the median (IQR) start day was 29 (20-44) days after birth for dexamethasone and 52 (30-90) days after birth for prednisolone or methylprednisolone. The median (IQR) total days of exposure was 10 (5-15) days for dexamethasone and 13 (6-25) days for prednisolone or methylprednisolone. There was minimal variation based on GA at birth regarding the start day or length of exposure (eTable 1 in Supplement 1). The median (IQR) cumulative dose for dexamethasone was 1.3 (0.9-2.7) mg/kg.

**Figure 2.**
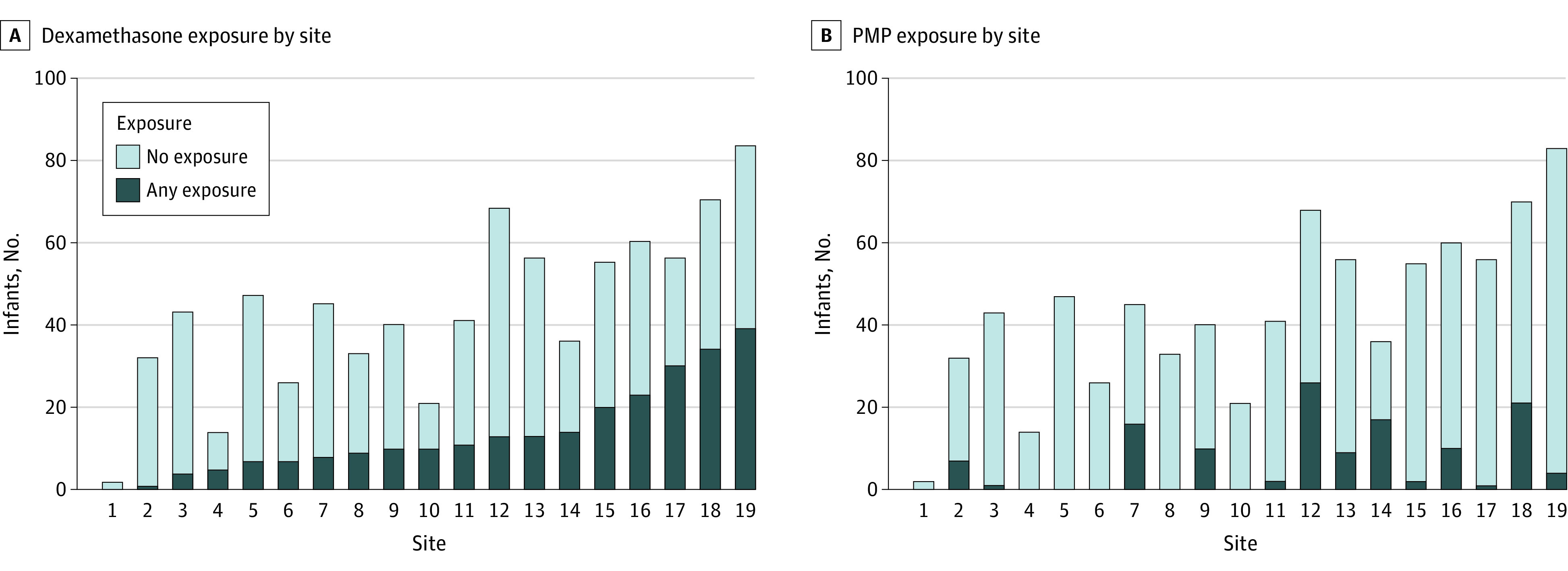
Infants Exposed to Dexamethasone and Prednisolone or Methylprednisolone (PMP) by Site and Steroid Type

Of 258 infants exposed to dexamethasone, 7 infants (2.7%) were exposed by day 7, 31 infants (12.0%) were exposed by day 14, 77 infants (19.8%) were exposed by day 21, and 126 infants (48.8%) were exposed by day 28 after birth. By comparison, of 126 infants exposed to prednisolone or methylprednisolone, 3 infants (2.4%) were exposed by day 7, 8 infants (6.3%) were exposed by day 14, 13 infants (10.3%) were exposed by day 21, 29 infants (23.0%) were exposed by day 28, and 70 infants (55.6%) were exposed by day 56 after birth. The use of corticosteroids varied across the 19 sites, with median (IQR) exposure of 27% (18%-39%) for dexamethasone and 5% (0%-23%) for prednisolone or methylprednisolone (Figure 2).

The median (IQR) number of dexamethasone courses per exposed infant was 1 (1-2; range 1-8). In 153 infants who received a single course, the median (IQR) duration was 5 (3-11) days (range, 2-39 days). In 105 infants who received more than 1 course, median (IQR) course length was 6 (4-9) days (range, 2-27 days).

The median (IQR) number of prednisolone or methylprednisolone courses per infant exposed was 1 (1-2; range 1-6). In 76 infants who received a single course, the median (IQR) duration was 8 (5-14) days (range, 2-71 days). In 50 infants who received more than 1 course, median (IQR) course length was 10 (7-19) days (range, 3-64 days).

### BSID-III Assessments at 2 Years Corrected Age

BSID-III assessments were conducted at a median (IQR) age of 24 (22-25) months for infants not exposed to corticosteroids. For infants who were exposed to dexamethasone or prednisolone or methylprednisolone, median (IQR) age at BSID-III assessment was 23 (22-25) months.

#### Dexamethasone

eFigure 2 in Supplement 1 shows the BSID-III cognitive, motor, and language scores by total days of exposure and by cumulative dose in milligrams per kilogram. Each additional day of exposure, compared with infants not exposed to corticosteroids, was associated with lower cognitive (adjusted mean difference, –0.22 [95% CI, –0.42 to –0.02] points) and motor (adjusted mean difference, –0.31 [95% CI, –0.54 to –0.08] points) scores. These results were robust to sensitivity analyses, with the associations remaining unchanged when additionally adjusted for total cumulative dose. There was no association of language scores with exposure (adjusted mean difference, –0.16 [95% CI, –0.42 to 0.09] points) (eTable 2 in Supplement 1). When examined by total cumulative dose, dexamethasone was not associated with a decline in BSID-III scores per milligram per kilogram of exposure.

The median (IQR) cumulative dose of dexamethasone exposure was similar among groups at 3 days (1.1 [0.8-1.7] mg/kg), 4 to 7 days (0.8 [0.6-2.0] mg/kg), and 8 to 14 days (1.1 [0.9-1.5] mg/kg) and 3-fold lower than in infants who received dexamethasone for longer than 14 days (3.1 [2.0-5.0] mg/kg). Prolonged exposure among 48 infants with more than 14 days exposure and 2-year outcome data was associated with lower motor (adjusted mean difference, –7.4 [95% CI, –12.3 to –2.5] points; *P* = .003) and language (adjusted mean difference, –5.8 [95% CI, –10.9 to –0.6] points; *P* = .03) scores after adjustment for treatment group, GA, SGA, respiratory support at 14 days, in-hospital SAEs, and maternal education (Table 2). Sensitivity analysis adjusting for clinical site found similar associations between longer than 14 days of dexamethasone exposure and reduced BSID-III scores across all 3 subscales (eTable 3 in Supplement 1).

**Table 2.** Adjusted BSID-III Cognitive, Motor, and Language Scores by Days of Exposure to Dexamethasone and PMP

Model	Cumulative dose		BSID-III subscore								
			Cognitive			Motor			Language		
	No.	Median (IQR), mg/kg	No.	AMD (95% CI)	*P* value	No.	AMD (95% CI)	*P* value	No.	AMD (95% CI)	*P* value
**Dexamethasone duration, d**											
Model 1^a^											
None	570	NA	485	0 [Reference]	NA	474	0 [Reference]	NA	473	0 [Reference]	NA
≤3	43	1.1 (0.8 to 1.7)	30	–4.5 (–10.9 to 1.9)	.16	29	–8.5 (–15.9 to –1.0)	.03	30	–6.1 (–13.3 to 1.0)	.09
4-7	57	0.8 (0.6 to 2.0)	47	–2.0 (–6.6 to 2.7)	.42	47	–4.2 (–9.0 to 0.5)	.08	46	–1.4 (–6.5 to 3.8)	.60
7-14	90	1.1 (0.9 to 1.5)	73	–2.6 (–6.8 to 1.5)	.22	73	–3.7 (–7.9 to 0.6)	.09	71	–0.8 (–5.7 to 4.2)	.76
>14	48	3.1 (2.0 to 5.0)	57	–7.6 (–12.1 to –3.1)	.001	57	–11.4 (–16.5 to –6.4)	<.001	57	–9.1 (–14.2 to –4.0)	<.001
Model 2^b^											
None	570	NA	485	0 [Reference]	NA	474	0 [Reference]	NA	473	0 [Reference]	NA
≤3	43	1.1 (0.8 to 1.7)	30	–3.2 (–9.5 to 3.1)	.32	29	–7.1 (–14.4 to 0.4)	.07	30	–4.6 (–11.7 to 2.4)	.20
4-7	57	0.8 (0.6 to 2.0)	47	0.06 (–4.6 to 4.7)	.98	47	–2.0 (–6.7 to 2.8)	.42	46	1.0 (–4.2 to 6.2)	.70
7-14	90	1.1 (0.9 to 1.5)	73	–0.4 (–4.8 to 3.9)	.84	73	–1.1 (–5.4 to 3.2)	.61	71	1.8 (–3.3 to 6.8)	.49
>14	48	3.1 (2.0 to 5.0)	57	–5.8 (–10.6 to –1.1)	.02	57	–9.4 (–14.6 to –4.3)	<.001	57	–7.1 (–12.4 to –1.7)	.001
Model 3^c^											
None	570	NA	485	0 [Reference]	NA	474	0 [Reference]	NA	473	0 [Reference]	NA
≤3	43	1.1 (0.8 to 1.7)	30	–2.0 (–8.3 to 4.2)	.52	29	–5.2 (–11.9 to 1.6)	.13	30	–3.2 (–10.5 to 4.0)	.38
4-7	57	0.8 (0.6 to 2.0)	47	0.2 (–4.2 to 4.7)	.92	47	–1.0 (–5.6 to 3.6)	.67	46	1.1 (–4.0 to 6.2)	.68
7-14	90	1.1 (0.9 to 1.5)	73	–0.7 (–5.2 to 3.7)	.75	73	–0.7 (–5.0 to 3.5)	.74	71	1.3 (–3.8 to 6.3)	.62
>14	48	3.1 (2.0 to 5.0)	57	–4.6 (–9.3 to 0.2)	.06	57	–7.4 (–12.3 to –2.5)	.003	57	–5.8 (–10.9 to –0.6)	.03
**PMP duration, d**											
Model 1^a^											
None	NA	NA	593	0 [Reference]	NA	585	0 [Reference]	NA	581	0 [Reference]	NA
≤7	NA	NA	32	–2.0 (–8.1 to 4.2)	.53	32	–3.6 (–11.3 to 4.1)	.36	30	0.4 (–7.5 to 8.3)	.92
8-14	NA	NA	24	4.2 (–0.9 to 9.4)	.11	22	4.1 (–1.4 to 9.5)	.14	23	6.4 (–0.7 to 13.5)	.08
15-28	NA	NA	24	–3.8 (–9.6 to 2.0)	.20	22	0.1 (–6.2 to 6.4)	.98	24	–0.8 (–7.0 to 5.3)	.79
>28	NA	NA	19	–0.5 (–7.4 to 6.5)	.89	19	–7.9 (–14.2 to –0.9)	**.03**	19	4.0 (–3.8 to 11.8)	.32
Model 2^b^											
None	NA	NA	593	0 [Reference]	NA	585	0 [Reference]	NA	581	0 [Reference]	NA
≤7	NA	NA	32	0.0 (–6.3 to 6.2)	.99	32	–1.2 (–8.9 to 6.6)	.77	30	2.4 (–5.7 to 10.4)	.56
8-14	NA	NA	24	6.1 (1.0 to 11.1)	.02	22	6.7 (1.4 to 12.0)	.01	23	8.7 (1.8 to 15.6)	.01
15-28	NA	NA	24	–0.2 (–5.8 to 5.4)	.94	22	4.5 (–0.9 to 10.0)	.10	24	2.2 (–4.3 to 8.6)	.51
>28	NA	NA	19	0.6 (–6.4 to 7.5)	.87	19	–6.6 (–13.4 to 0.2)	.06	19	5.1 (–2.5 to 12.7)	.19
Model 3^c^											
None	NA	NA	593	0 [Reference]	NA	585	0 [Reference]	NA	581	0 [Reference]	NA
≤7	NA	NA	32	0.3 (–6.3 to 6.8)	.94	32	0.3 (–7.7 to 8.4)	.94	30	2.6 (–5.6 to 10.7)	.53
8-14	NA	NA	24	5.5 (0.1 to 10.9)	.05	22	5.9 (0.4 to 11.4)	.04	23	9.2 (2.6 to 15.8)	.006
15-28	NA	NA	24	0.5 (–5.0 to 6.0)	.87	22	5.7 (0.6 to 10.7)	.03	24	4.3 (–2.3 to 10.9)	.20
>28	NA	NA	19	0.9 (–6.1 to 7.9)	.80	19	–4.9 (–11.9 to 2.1)	.17	19	4.9 (–2.8 to 12.6)	.21

Abbreviations: AMD, adjusted mean difference; BSID-III, Bayley Scales of Infant Development–Third Edition; NA, not applicable; PMP, prednisolone or methylprednisolone.

^a^ Adjusted for treatment group (erythropoietin/placebo) and gestational age in weeks.

^b^ Additionally adjusted for SGA and respiratory support at 14 days (none or nasal cannula, positive pressure, or intubated).

^c^ Further adjusted to account for in-hospital severe adverse events associated with outcomes (necrotizing enterocolitis, severe intraventricular hemorrhage, retinopathy of prematurity, spontaneous intestinal perforation, and severe bronchopulmonary dysplasia), as well as maternal education to improve the accuracy of the estimated effect of exposure.

#### Prednisolone or Methylprednisolone

eFigure 2 in Supplement 1 shows the BSID-III cognitive, motor, and language scores by total days of exposure. In infants exposed to prednisolone or methylprednisolone, no linear per-day associations of treatment were observed in BSID-III cognitive, motor, or language scores. Exposure to prednisolone or methylprednisolone for more than 28 days was associated with lower motor scores (adjusted mean difference, –7.9 [95% CI, –14.2 to –0.9] points) (Table 2). With additional adjustments for SGA, respiratory support at 14 days, maternal education, and SAEs, length of exposure was no longer associated with motor outcomes. In the fully adjusted model 3, all BSID-III subscale scores were higher in infants with 8 to 14 days exposure compared with infants with no corticosteroid exposure (Table 2). There was no linear association between prednisolone or methylprednisolone exposure duration and BSID-III scores at corrected age 2 years (eTable 2 in Supplement 1).

### CP

Documentation of CP status was available in 700 infants (84.5%), of whom 85 infants (12.1%) had CP documented. Of 85 infants with CP, 39 (45.9%) had been exposed to dexamethasone, 15 (17.6%) had been exposed to prednisolone or methylprednisolone, and 9 (10.6%) had been exposed to both. Overall, 46 of 490 infants (9.4%) of not exposed to dexamethasone developed CP, compared with 39 of 210 infants (18.6%) with any dexamethasone exposure, 15 of 99 infants (15.2%) with any prednisolone or methylprednisolone exposure, and 9 of 52 infants (17.3%) exposed to both. Exposure to the corticosteroids of interest was not significantly associated with increased odds of CP after adjusting for GA, treatment group, SGA status, respiratory support at 14 days, maternal education, and SAEs (OR, 1.5; 95% CI, 0.8 to 2.8; *P* = .23). Of 39 infants with CP and dexamethasone exposure, 27 (69.2%) had mild CP and 12 (30.8%) had moderate-severe CP. Of 15 infants with CP and prednisolone or methylprednisolone exposure, 10 (66.7%) had mild CP and 5 (33.3%) had moderate-severe CP.

## Discussion

In this multicenter cohort study of extremely preterm infants with multiple adjustments, we found that using dexamethasone for more than 14 days during the initial NICU stay was associated with lower BSID-III motor and language scores at corrected age 2 years. For infants exposed to dexamethasone, each additional day of dexamethasone exposure was associated with significantly lower cognitive and motor scores but not language scores. For infants exposed to prednisolone or methylprednisolone, exposure for more than 28 days was associated with lower motor scores; however, after additional adjustments, the days of exposure to prednisolone or methylprednisolone were no longer associated with lower motor scores; cognitive, motor, and language scores in the 8 to 14 days exposure group and motor scores in the 15 to 28 days exposure group were higher compared with the unexposed group. There was no linear per-day association of prednisolone or methylprednisolone treatment observed in BSID-III scores.

One third of extremely preterm infants enrolled in our cohort were treated with dexamethasone, and most of them were exposed after the first week of life. The baseline infant characteristics, exposure rate, and timing of exposure are consistent with other recent studies in Europe.^29^ However, our findings highlight the variability of exposure to dexamethasone across NICUs, with a range from 0% to 50% among 19 PENUT sites. This is consistent with previous reports showing considerable practice variation among hospitals, even for infants with similar characteristics and illness severity (3%-50%).^29,38^

We used timing, total cumulative dose, and duration of treatment to describe dexamethasone exposure and its association with neurodevelopmental outcomes. Prior data from randomized clinical trials evaluating the effect of dexamethasone on neurodevelopment vary, from no effect to severe disability.^21,25,39,40,41,42^ Although early exposure (age <7 days) has been associated with decreased BPD rates in some studies, it has also been associated with an increased risk of both in-hospital complications as well as neurodevelopmental impairment and CP.^8,15,22^ The associations between later exposure (age >7 days) and neurodevelopmental outcomes are not conclusive, with emerging support for the use of dexamethasone in infants at high risk of developing BPD.^9^

Short exposure to dexamethasone in this cohort was not significantly associated with BSID-III scores at corrected age 2 years. Our findings were consistent with 2 other randomized clinical trials evaluating the impact of low dose or short exposure to dexamethasone (0.15 mg/kg/d for 3 days, then tapering over 7 days) that did not find a significant increase in CP or neurodevelopmental impairment compared with placebo.^15,41^

The incidence of CP in this study was 19% of infants with dexamethasone exposure compared with 9% in infants with no corticosteroid exposure. In a study by Wilson-Costello et al^43^ in which infants were exposed to corticosteroids for longer periods (up to 75 days) and at higher doses than infants in the PENUT trial, every 1-mg/kg increase in the cumulative dose of dexamethasone was associated with a 40% increased risk of developing disabling CP at every GA.^43^

While we do not recommend a specific dexamethasone regimen based on the results from our cohort, we found that higher cumulative doses and longer exposures (>14 days) were negatively associated with 2-year neurodevelopmental outcomes. After adjustments for treatment group, GA at birth, SGA status, respiratory support at age 2 weeks, SAEs, and maternal education, BSID-III motor scores showed the biggest difference vs infants with no exposure. The Canadian Pediatric Society suggested in 2020 that clinicians consider a short course of low-dose dexamethasone to prevent or treat BPD (0.15 mg/kg/d to 0.2 mg/kg/d tapered over 7-12 days).^28^ In agreement with this recommendation, Ramaswamy et al^30^ performed a systematic review and network meta-analysis to compare the outcomes and safety of 14 postnatal corticosteroid regimens and concluded that moderately early initiation (8-14 days after birth), medium cumulative dose (2-4 mg/kg), and short duration (<8 days) of systemic dexamethasone might be the most appropriate regimen for reducing the risk of BPD or mortality at postmenstrual age 36 weeks.^30^ In a commentary on the study by Ramaswamy et al,^30^ Parikh^44^ called for more studies evaluating neurodevelopmental impairment outcomes of high-dose dexamethasone regimens before changing clinical practice.

Although most infants at high risk for developing BPD receive dexamethasone, there is emerging data to support alternative systemic or inhaled corticosteroid therapies that may not carry the same neurodevelopmental sequelae burden.^28,45^ Our study evaluating the association between the days of exposure to prednisolone or methylprednisolone and BSID-III scores at corrected age 2 years found an association between prolonged (>28 days) exposure and BSID-III motor scores that was not present after additional adjustments for SGA, respiratory support at 14 days, maternal education, and SAEs. We can speculate that an association between prednisolone or methylprednisolone exposure and BSID-III scores was not observed in our cohort owing to the smaller number of infants who received prednisolone or methylprednisolone and the more advanced age and more mature brains at the time of exposure. Further studies designed to evaluate the association between other classes of corticosteroids and neurodevelopment are needed.

### Limitations

Our study has several limitations. First, the PENUT trial was not designed to primarily evaluate the impact of dexamethasone or prednisolone or methylprednisolone on 2-year neurodevelopmental outcomes. Although we adjusted for known potential confounders, there may still be residual confounding by indication. Infants received the medications based on the attending physician’s clinical judgment, which is reflected in the different clinical characteristics of the groups and the variability among the sites. The indications for use of corticosteroids in each infant were not available. A significant limitation is the lack of data in this data set denoting the exact timing of invasive ventilation and respiratory support weaning strategies, as well as risk for BPD at the time of steroid administration. Additionally, we did not perform an analysis to evaluate the timing of postnatal systemic steroid exposure in association with any morbidity or adverse event. Owing to the relatively small number of infants within each level of prednisolone or methylprednisolone exposure, it is possible that the analysis was underpowered to detect a true association of exposure with outcome. Additionally, our findings do not reflect causal associations and may be due to unmeasured factors associated with exposure to dexamethasone or prednisolone or methylprednisolone and infant neurodevelopmental outcomes.

## Conclusions

This cohort study found that prolonged dexamethasone treatment was associated with lower BSID-III scores at age 2 years. Each additional day of dexamethasone exposure was associated with significantly lower cognitive and motor scores. There were no linear associations between dexamethasone dose or prednisolone or methylprednisolone exposure duration and BSID-III scores at age 2 years. Prospective studies and longer-term outcomes, including school-age evaluations, are necessary to inform and guide the use of postnatal steroids in this high-risk population.

## Notes

Supplement 1. **eFigure 1.** Association Between Days of Dexamethasone Exposure and BSID-III Motor Score **eFigure 2.** BSID-III Cognitive, Motor, and Language Scores by Days of Exposure and Cumulative Dose for Dexamethasone and by Days of Exposure to Prednisolone or Methylprednisolone **eTable 1.** Days of Exposure and Start Day for Dexamethasone and Prednisolone or Methylprednisolone Overall and by Gestational Age **eTable 2.** BSID-III Cognitive, Motor, and Language Scores by Days of Exposure and Cumulative Dose of Dexamethasone and Prednisolone or Methylprednisolone **eTable 3.** Sensitivity Analysis Adjusting for Clinical Site

Supplement 2.
Members of the Preterm Erythropoietin Neuroprotection (PENUT) Trial Consortium
