# Supplementary material for: Dexamethasone, Prednisolone, and Methylprednisolone Use and 2-Year Neurodevelopmental Outcomes in Extremely Preterm Infants
Source: JAMA Netw Open. 2022 Mar 11;5(3):e221947. doi: 10.1001/jamanetworkopen.2022.1947 (PMC8917427; doi:10.1001/jamanetworkopen.2022.1947)
Supplement: Supplement 1. — eFigure 1. Association Between Days of Dexamethasone Exposure and BSID-III Motor Score eFigure 2. BSID-III Cognitive, Motor, and Language Scores by Days of Exposure and Cumulative Dose for Dexamethasone and by Days of Exposure to Prednisolone or Methylprednisolone eTable 1. Days of Exposure and Start Day for Dexamethasone and Prednisolone or Methylprednisolone Overall and by Gestational Age eTable 2. BSID-III Cognitive, Motor, and Language Scores by Days of Exposure and Cumulative Dose of Dexamethasone and Prednisolone or Methylprednisolone eTable 3. Sensitivity Analysis Adjusting for Clinical Site [file jamanetwopen-e221947-s001.pdf]

Supplemental Online Content

Puia-Dumitrescu M, Wood TR, Comstock BA, et al; Preterm Erythropoietin Neuroprotection PENUT Trial Consortium. Dexamethasone, prednisolone, and methylprednisolone use and 2-year neurodevelopmental outcomes in extremely preterm infants. *JAMA Netw Open*. 2022;5(3):e221947. doi:10.1001/jamanetworkopen.2022.1947

eFigure 1. Association Between Days of Dexamethasone Exposure and BSID-III Motor Score
eFigure 2. BSID-III Cognitive, Motor, and Language Scores by Days of Exposure and Cumulative Dose for Dexamethasone and by Days of Exposure to Prednisolone or Methylprednisolone
eTable 1. Days of Exposure and Start Day for Dexamethasone and Prednisolone or Methylprednisolone Overall and by Gestational Age
eTable 2. BSID-III Cognitive, Motor, and Language Scores by Days of Exposure and Cumulative Dose of Dexamethasone and Prednisolone or Methylprednisolone
eTable 3. Sensitivity Analysis Adjusting for Clinical Site

This supplemental material has been provided by the authors to give readers additional information about their work.

© 2022 Puia-Dumitrescu M et al. *JAMA Network Open.*

eFigure 1. Association Between Days of Dexamethasone Exposure and BSID-III Motor Score

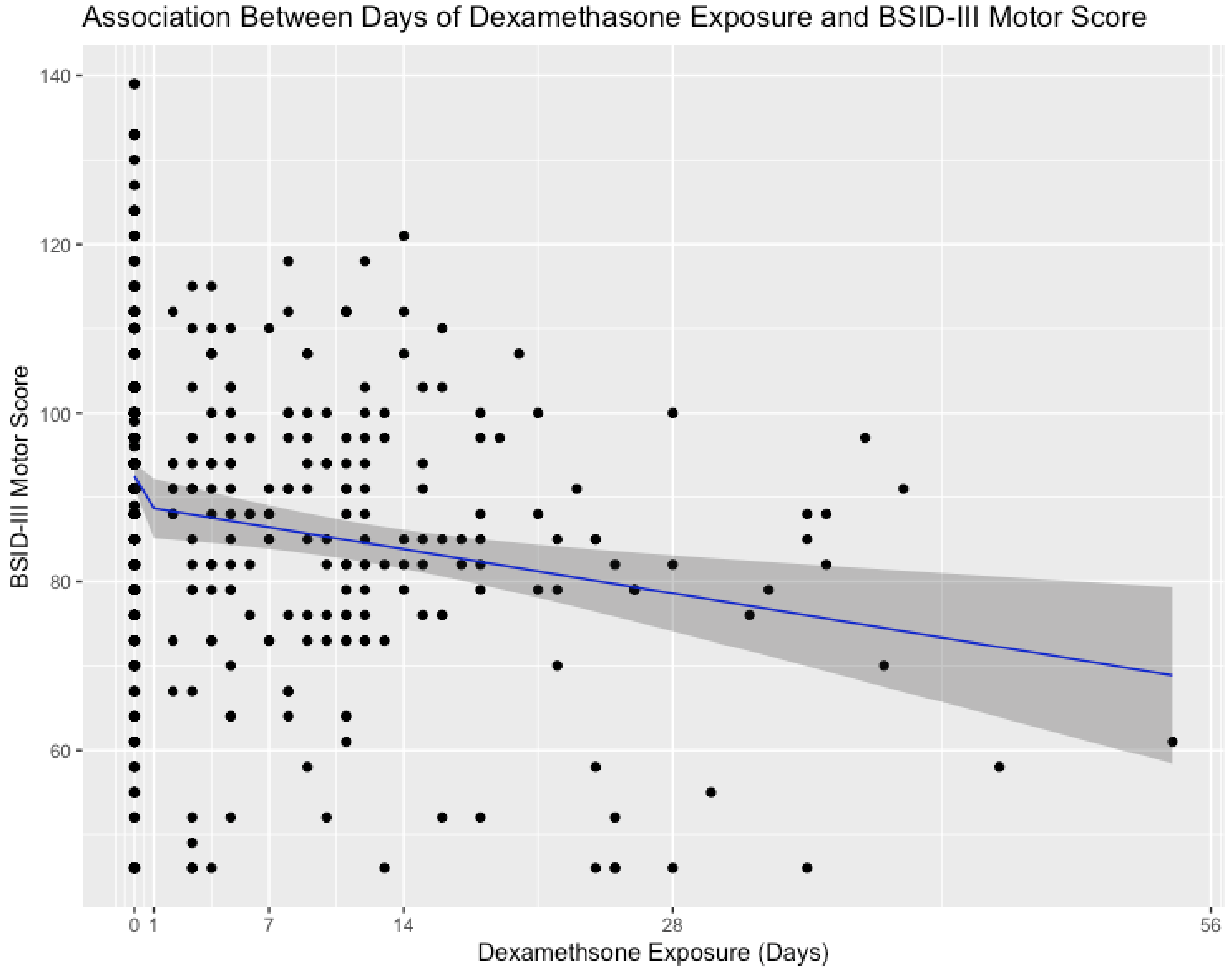

Unadjusted linear spline (with 95%CI) showing a proposed relationship between days of exposure to dexamethasone and BSID-III Motor Score, with a single knot at 1 day of exposure. The results of this model are shown in eTable 2.

© 2022 Puia-Dumitrescu M et al. *JAMA Network Open.*

eFigure 2. BSID-III Cognitive, Motor, and Language Scores by Days of Exposure and Cumulative Dose for Dexamethasone and by Days of Exposure to Prednisolone or Methylprednisolone

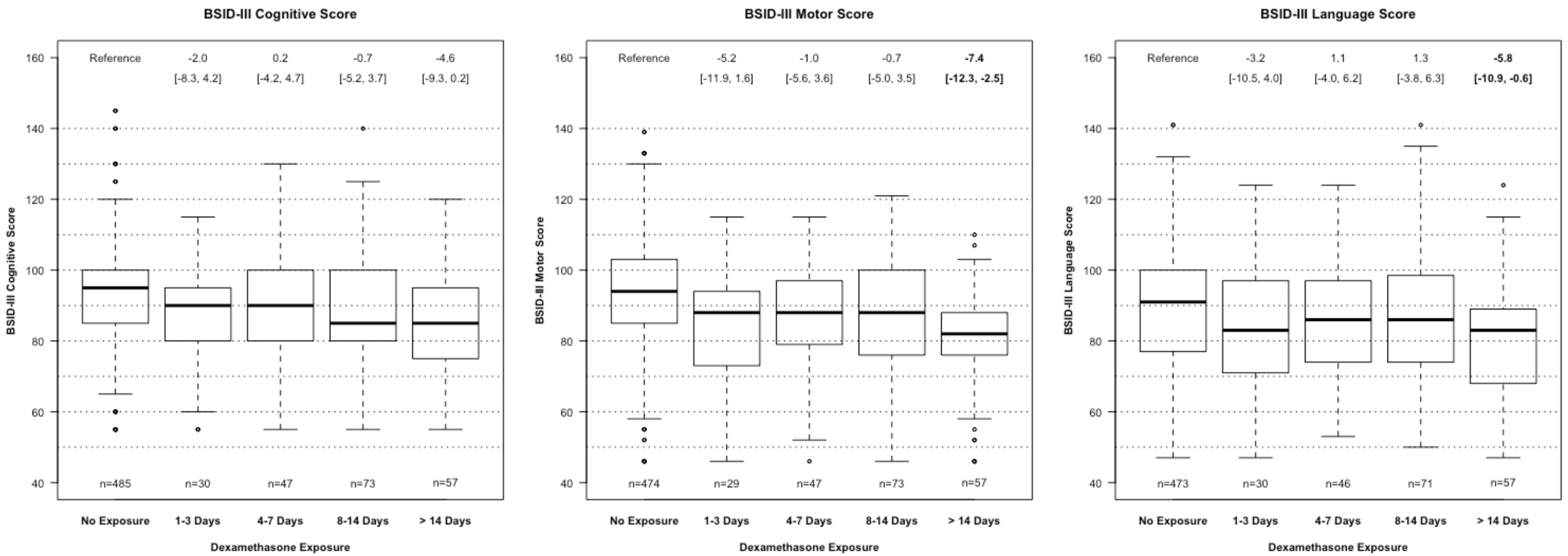

eFigure 2A. BSID-III cognitive, motor, and language scores by days of exposure to dexamethasone. Box plot of median (interquartile range) cognitive, motor, and language scores at 2 years' corrected age by exposure to dexamethasone defined as total number of days of exposure. Boxes contain 50% of data, with the inside horizontal line representing the median value; whiskers contain 100% of data, except for statistical outliers, which are shown as individual data points. Values above each box indicate mean difference in that subscale (with 95% CI) for effect of that exposure level relative to unexposed infants in the fully adjusted model 3. Bold indicates 95% CI that do not cross 0.

© 2022 Puia-Dumitrescu M et al. *JAMA Network Open.*

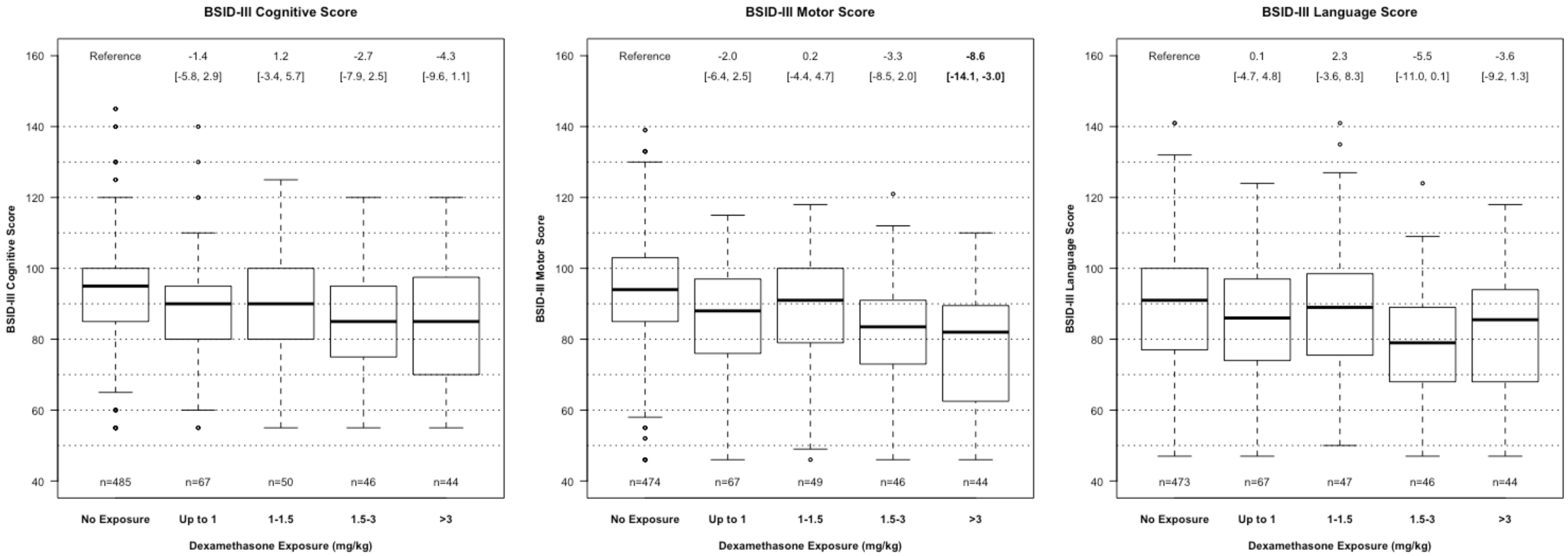

eFigure 2B. BSID-III cognitive, motor, and language scores by cumulative dose (mg/kg) of dexamethasone. Box plot of median (interquartile range) cognitive, motor, and language scores at 2 years' corrected age by exposure to dexamethasone defined as total cumulative dose. Boxes contain 50% of data, with the inside horizontal line representing the median value; whiskers contain 100% of data, except for statistical outliers, which are shown as individual data points. Values above each box indicate mean difference in that subscale (with 95% CI) for effect of that exposure level relative to unexposed infants in the fully adjusted model 3. Bold indicates 95% CI that do not cross 0.

© 2022 Puia-Dumitrescu M et al. *JAMA Network Open.*

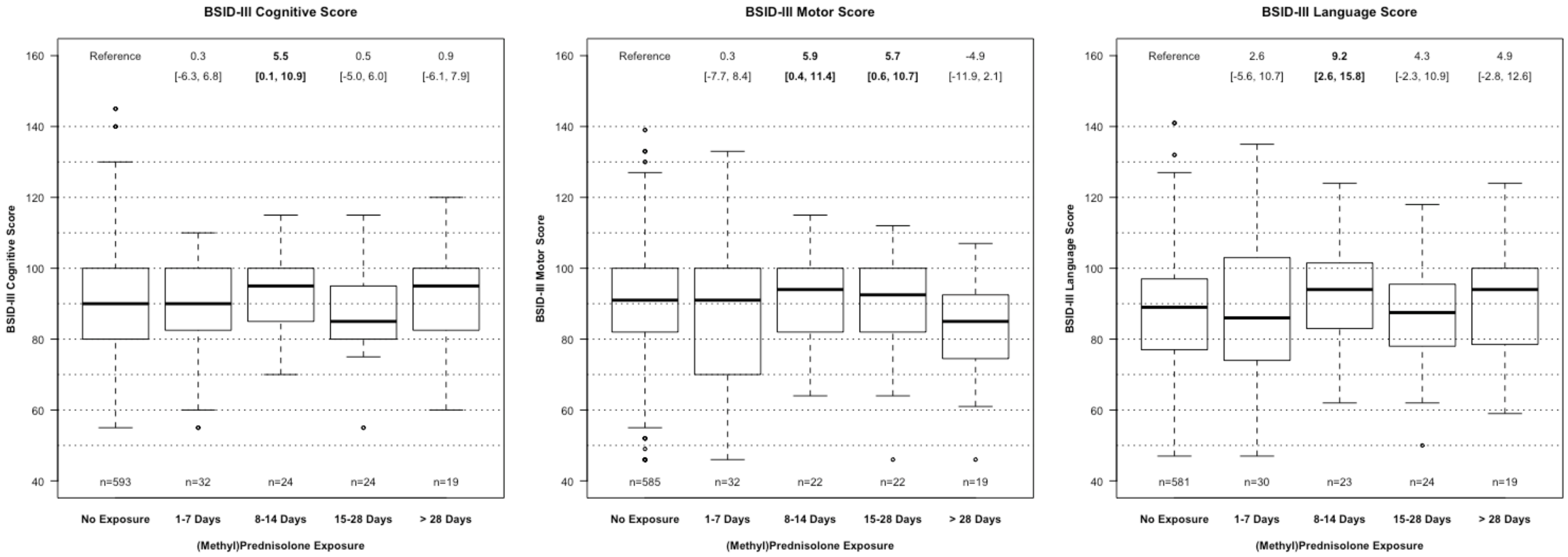

eFigure 2C. BSID-III cognitive, motor, and language scores by days of exposure to (methyl)prednisolone. Box plot of median (interquartile range) cognitive, motor, and language scores at 2 years' corrected age by total number of days of exposure to (methyl)prednisolone. Boxes contain 50% of data, with the inside horizontal line representing the median value; whiskers contain 100% of data, except for statistical outliers, which are shown as individual data points. Values above each box indicate mean difference in that subscale (with 95% CI) for effect of that exposure level relative to unexposed infants in the fully adjusted model 3. Bold indicates 95% CI that do not cross 0.

© 2022 Puia-Dumitrescu M et al. *JAMA Network Open.*

eTable 1. Days of Exposure and Start Day for Dexamethasone and Prednisolone or Methylprednisolone Overall and by Gestational Age
Number of available infants for each analysis/outcome is provided (n=).

	Dexamethasone			(Methyl)Prednisolone		
	**n=**	**Median (IQR)**	**Range**	**n=**	**Median (IQR)**	**Range**
**Days of exposure**						
Overall	258	10 (5-15)	2-104	126	13 (6-25)	2-175
24 weeks	104	11 (5-18)	2-84	49	13 (7-23)	2-138
25 weeks	79	10 (4-13)	2-45	33	11 (6-23)	2-142
26 weeks	43	10 (5-13)	2-54	21	15 (8-22)	2-88
27 weeks	32	7 (4-14)	2-104	23	20 (5-38)	2-175
**Start day**						
Overall	258	29 (20-44)	2-175	126	53 (30-90)	2-234
24 weeks	104	28 (20-41)	2-174	49	56 (38-95)	2-234
25 weeks	79	30 (21-43)	5-139	33	53 (38-93)	10-183
26 weeks	43	29 (20-45)	4-175	21	37 (25-77)	13-161
27 weeks	32	38 (24-69)	4-169	23	44 (27-69)	3-102

© 2022 Puia-Dumitrescu M et al. *JAMA Network Open.*

eTable 2. BSID-III Cognitive, Motor, and Language Scores by Days of Exposure and Cumulative Dose of Dexamethasone and Prednisolone or Methylprednisolone

Coefficient (coef) is the adjusted mean difference in that subscale score associated with each additional day of exposure in exposed infants (see Supplemental Figure 2 for model structure). Three different models were developed for effect of exposure by length of exposure; model 1 was adjusted for treatment group (Epo/placebo) and GA in weeks; model 2 was additionally adjusted for potential confounders associated with both exposure and outcome; model 3 was then further adjusted for maternal education and in-hospital severe adverse events that predict outcome in order to improve the accuracy of the estimated effect of exposure.

		**BSID-III Subscale - Adjusted Mean Score Difference Per Day of Exposure**					
		**Cognitive**		**Motor**		**Language**	
	**Model**	**Coef (95% CI)**	**p-value**	**Coef (95% CI)**	**p-value**	**Coef (95% CI)**	**p-value**
**Per day (Dex)**	**1**	-0.26 (-0.48, -0.04)	**0.02**	-0.35 (-0.60, -0.11)	**0.005**	-0.21 (-0.48, 0.06)	0.13
	**2**	-0.25 (-0.47, -0.03)	**0.02**	-0.34 (-0.58, -0.10)	**0.006**	-0.20 (-0.46, 0.07)	0.15
	**3**	-0.22 (-0.42, -0.02)	**0.03**	-0.31 (-0.54, -0.08)	**0.009**	-0.16 (-0.42, 0.09)	0.22

		**BSID-III Subscale - Adjusted Mean Score Difference Relative to Unexposed Infants**					
		**Cognitive**		**Motor**		**Language**	
	**Model**	**Coef (95% CI)**	**p-value**	**Coef (95% CI)**	**p-value**	**Coef (95% CI)**	**p-value**
**Per day (Pred)**	**1**	-0.00 (-0.11, 0.10)	0.95	-0.09 (-0.22, 0.03)	0.12	0.03 (-0.09, 0.15)	0.61
	**2**	-0.01 (-0.12, 0.10)	0.90	-0.10 (-0.23, 0.02)	0.10	0.03 (-0.09, 0.14)	0.67
	**3**	-0.01 (-0.13, 0.10)	0.82	-0.10 (-0.23, 0.02)	0.11	0.01 (-0.12, 0.14)	0.86

© 2022 Puia-Dumitrescu M et al. *JAMA Network Open.*

eTable 3. Sensitivity Analysis Adjusting for Clinical Site

Adjusted BSID III cognitive, motor and language scores by days of exposure to dexamethasone. Coefficient (coef) is the adjusted mean difference in that subscale score associated with that level of steroid exposure, compared to unexposed infants. Number of available infants for each analysis/outcome is provided (n=). In addition to the fully adjusted model 3, additional adjustment is provided for each of the 19 clinical sites.

		BSID-III Subscales - Adjusted Mean Score Difference Relative to Unexposed Infants								
		Cognitive			Motor			Language		
**Adjustment**		**n=**	**Coef (95% CI)**	**p-value**	**n=**	**Coef (95% CI)**	**p-value**	**n=**	**Coef (95% CI)**	**p-value**
**Model 3 + Clinical Site**	**No Dex**	485	Reference	-	474	Reference	-	473	Reference	-
	**Up to 3 days**	30	-1.7 (-7.8, 4.4)	0.59	29	-4.8 (-11.5, 1.9)	0.16	30	-2.6 (-9.6, 4.4)	0.46
	**4-7 days**	47	-0.7 (-5.0, 3.7)	0.76	47	-1.3 (-5.5, 3.0)	0.56	46	0.0 (-5.0, 5.0)	0.99
	**7-14 days**	73	-1.6 (-5.9, 2.6)	0.46	73	-1.6 (-5.7, 2.5)	0.45	71	1.0 (-3.5, 5.6)	0.65
	**>14 days**	57	-6.1 (-11.0, -1.2)	**0.01**	57	-8.8 (-14.0, -3.7)	**<0.001**	57	-6.6 (-11.4, -1.7)	**0.008**

© 2022 Puia-Dumitrescu M et al. *JAMA Network Open.*
