# Supplementary material for: Dexamethasone, Prednisolone, and Methylprednisolone Use and 2-Year Neurodevelopmental Outcomes in Extremely Preterm Infants
Source: JAMA Netw Open. 2022 Mar 11;5(3):e221947. doi: 10.1001/jamanetworkopen.2022.1947 (PMC8917427; doi:10.1001/jamanetworkopen.2022.1947)
Supplement: Supplement 2. — Members of the Preterm Erythropoietin Neuroprotection (PENUT) Trial Consortium [file jamanetwopen-e221947-s002.pdf]

Supplemental Online Content: Nonauthor Collaborators

*Indicates required information. Only first name, last name, and suffix will appear in PubMed.

*Group Name(s): Preterm Erythropoietin Neuroprotection (PENUT) Trial Consortium							
***First Name and Middle Initial(s)**	***Last Name**	***Suffix (eg, Jr, III)**	Academic Degrees	Institution	Location (city, state/province, country)	Role or Contribution, eg, chair, principal investigator	Group (if more than 1 Group listed in the byline) and/or Subgroup (eg, Steering Committee)
Rajan	Wadhawan		MD	Advent Health for Children	Orlando, Florida	PENUT Site PI	
Sherry E	Courtney		MD	University of Arkansas for Medi	Little Rock, Arkansas	PENUT Site PI	
Tonya	Robinson		MD	University of Louisville	Louisville, Kentucky	PENUT Site PI	
Kaashif A	Ahmad		MD MSc	Methodist Children's Hospital	San Antonio, Texas	PENUT Site PI	
Ellen	Bendel-Stenzel		MD	Children's Minnesota	Minneapolis, Minnesota	PENUT Site PI	
Mariana	Baserga		MD	University of Utah	Salt Lake City, Utah	PENUT Site PI	
Edmund F	LaGamma		MD	Maria Fareri Children's Hospita	Valhalla, New York	PENUT Site PI	
L Corbin	Downey		MD	Wake Forest School of Medicir	Winston-Salem, North	PENUT Site PI	
Raghavendra	Rao		MD	University of Minnesota Mason	Minneapolis, Minnesota	PENUT Site PI	
Nancy	Fahim		MD MSc	University of Minnesota Mason	Minneapolis, Minnesota	PENUT Site PI	
Andrea	Lampland		MD	Children's Minnesota	St. Paul, Minnesota	PENUT Site PI	
Ivan D	Frantz	III	MD	Beth Israel Deaconess Medical	Boston, Massachusetts	PENUT Site PI	
Janine	Khan		MD	Prentice Women's Hospital	Chicago, Illinois	PENUT Site PI	
Michael	Weiss		MD	University of Florida	Gainesville, Florida	PENUT Site PI	
Maureen M	Gilmore		MD	Johns Hopkins University	Baltimore, Maryland	PENUT Site PI	
Robin K	Ohls		MD	University of New Mexico	Albuquerque, New Mex	PENUT Site PI	
Jean	Lowe		PhD	University of New Mexico	Albuquerque, New Mex	PENUT Site PI	
Nishant	Srinivasan		MD	Children's Hospital of the Unive	Chicago, Illinois	PENUT Site PI	
Jorge E	Perez		MD	South Miami Hospital	South Miami, Florida	PENUT Site PI	
Victor	McKay		MD	Johns Hopkins All Children's H	St. Petersburg, Florida	PENUT Site PI	
